# Prescription Medications and Co-Morbidities in Late Middle-Age are Associated with Greater Cognitive Declines: Results from WRAP

**DOI:** 10.3389/fragi.2021.759695

**Published:** 2022-01-03

**Authors:** Lianlian Du, Rebecca Langhough Koscik, Nathaniel A. Chin, Lisa C. Bratzke, Karly Cody, Claire M. Erickson, Erin Jonaitis, Kimberly D. Mueller, Bruce P. Hermann, Sterling C. Johnson

**Affiliations:** ^1^ Wisconsin Alzheimer’s Institute, University of Wisconsin-Madison School of Medicine and Public Health, Madison, WI, United States; ^2^ Department of Biostatistics and Medical Informatics, School of Medicine and Public Health, University of Wisconsin-Madison, Madison, WI, United States; ^3^ Wisconsin Alzheimer’s Disease Research Center, Madison, WI, United States; ^4^ Department of Medicine, University of Wisconsin-Madison School of Medicine and Public Health, Madison, WI, United States; ^5^ School of Nursing, University of Wisconsin—Madison, Madison, WI, United States; ^6^ Department of Communication Sciences & Disorders, University of Wisconsin—Madison, Madison, WI, United States; ^7^ Department of Neurology, University of Wisconsin-Madison School of Medicine and Public Health, Madison, WI, United States; ^8^ Madison VA GRECC, William S. Middleton Memorial Hospital, Madison, WI, United States

**Keywords:** Alzheimer’s disease, mild cognitive impairment, polypharmacy, co-morbidities, executive function, PACC3, longitudinal cognitive performance

## Abstract

The present study investigated: 1) sex differences in polypharmacy, comorbidities, self-rated current health (SRH), and cognitive performance, 2) associations between comorbidities, polypharmacy, SRH, and objective measures of health, and 3) associations of these factors with longitudinal cognitive performance. Analyses included 1039 eligible Wisconsin Registry for Alzheimer’s Prevention (WRAP) participants who were cognitively unimpaired at baseline and had ≥2 visits with cognitive composites, self-reported health history, and concurrent medication records. Repeated measures correlation (rmcorr) examined the associations between medications, co-morbidities, SRH, and objective measures of health (including LIfestyle for BRAin Health Index (LIBRA), and depression). Linear mixed-effect models examined associations between medications, co-morbidities, and cognitive change over time using a preclinical Alzheimer’s cognitive composite (PACC3) and cognitive domain z-scores (executive function, working memory, immediate learning, and delayed recall). In secondary analyses, we also examined whether the number of medications interacted with co-morbidities and whether they modified age-related cognitive trajectories. The number of prescribed medications was associated with worse SRH and a higher number of self-reported co-morbidities. More prescribed medications were associated with a faster decline in executive function, and more comorbidities were associated with faster PACC3 decline. Those with a non-elevated number of co-morbidities and medications performed an average of 0.26 SD higher (better) in executive function and an average of 0.18 SD higher on PACC3 than those elevated on both. Associations between medications, co-morbidities, and executive function, and PACC3 suggest that persons with more co-morbidities and medications may be at increased risk of reaching clinical levels of impairment earlier than healthier, less medicated peers.

## Introduction

Polypharmacy, defined as taking five or more medications (Mortazavi et al., 2016), and medical co-morbidity, defined as multiple chronic conditions, are both associated with adverse effects on cognition, functional ability, and survival of individuals with dementia (Clague et al., 2017). Aging is associated with the accumulation of multiple co-morbidities and physical, cognitive, and psychological changes. Prescription medicines are still the most common form of medical intervention to manage chronic diseases, and polypharmacy is increasingly common among the elderly. For example, The National Center for Health Statistics (NCHS) recently reported that around 1 in 5 had polypharmacy (US 2015-2016, 22.4%; Canada 2016-2017, 18.8%) (Hales et al., 2019). This high rate of polypharmacy is driven in large part by people with multiple chronic medical conditions. Approximately half of the US population has at least one chronic condition, and one in four have multiple conditions (Ward et al., 2014). Currently, two-thirds of Alzheimer’s disease (AD) patients are females (Rahman et al., 2019).

Many AD risk factors show sex effects, with female sex being more severely impacted (Rahman et al., 2019). However, results were varied (Mielke et al., 2014; Livingston et al., 2017). People with AD take statistically significantly higher numbers of medication compared to controls even after age, sex, and comorbidity adjustment. In particular, previous work (Guthrie et al., 2010) showed that people with dementia are 17 times more likely to be prescribed an antipsychotic and twice as likely to be prescribed an antidepressant or a hypnotic/anxiolytic than older people without dementia. Some cross-sectional and longitudinal studies have suggested that polypharmacy is associated with cognitive decline and memory loss (Cheng et al., 2018; Khezrian et al., 2019; Soysal et al., 2019; Umegaki et al., 2019) and the same association was demonstrated among African American (AA) older adults (Assari et al., 2020). Previous research with cognitively unimpaired aged ≥70 years individuals also demonstrated that the risk of mild cognitive impairment (MCI) or AD was higher in individuals with multimorbidity, particularly in those with 4 + chronic conditions (Vassilaki et al., 2015). Also, faster accumulation of chronic diseases over time has been associated with faster cognitive decline (Fabbri et al., 2016), with multimorbidity furthermore cross-sectionally associated with imaging biomarkers of neurodegeneration in those aged ≥70 years (Vassilaki et al., 2016; Vemuri et al., 2017; Vassilaki et al., 2019).

However, few studies have extended this line of research in preclinical research, and few studies examining sex differences in cognition have controlled for concurrent medications and medical co-morbidity. More research is needed to better understand how cognition differs between women and men, and the association between polypharmacy, comorbidities, and cognition among people who do not have clinical levels of cognitive impairment. In addition, many previous studies have been limited by cross-sectional design or shorter follow-ups. Therefore, analyzing the associations between polypharmacy, co-morbidities, and longitudinal cognitive performance in a preclinical cohort with longer follow-up is important and may have implications for clinicians and patients. Using latent class analysis, our previous findings offered evidence of an association between the sleep class derived from self-reported multi-morbidities and the development of amnestic mild cognitive impairment (Bratzke et al., 2018), but this study didn’t include medication information, and the association between co-morbidities and different cognitive domains is not clear. To address these important gaps in our understanding of cognitive decline prior to clinical impairment, and follow the National Institutes of Health policy integrating sex as a biological variable (SABV) into biomedical research, the present study aims to investigate: 1) sex differences in polypharmacy, comorbidities, self-rated current health (SRH), and cognitive performance, 2) the associations between comorbidities, polypharmacy, SRH, and objective measures of health; and 3) the associations between polypharmacy, co-morbidities and longitudinal cognitive performance in the Wisconsin Registry for Alzheimer’s Prevention (WRAP; a longitudinal cohort risk-enriched for Alzheimer’s disease (AD) but non-demented at baseline). We hypothesized that more medications and co-morbidities would be associated with poorer SRH and poorer cognitive performance.

## Methods

### Participants

The WRAP cohort includes neuropsychological data from 1606 participants who enrolled at midlife (at baseline: mean age (sd) = 54.4 (6.7); 94.3% between ∼40-65 years of age; 73.3% with a parental family history of AD) and were free of dementia at baseline (Sager et al., 2005; Johnson et al., 2018). The study is designed to identify early cognitive decline and to characterize midlife factors (e.g., AD biomarkers, health and lifestyle factors) associated with such decline. Participant enrollment began in 2001; follow-up assessments with second-wave assessments were conducted approximately 2-4 years after baseline and all subsequent wave follow-up visits were conducted at approximately 2-year intervals. Participant retention, reported in (Johnson et al., 2018), was approximately 81% and median follow-up at that time was 9 years for active participants. This ongoing study is conducted in compliance with ethical principles for human subjects research defined in the Declaration of Helsinki, including review and approval by the University of Wisconsin Institutional Review Board, and the provision of informed consent by all participants.

For this study, all participants with complete data of interest (i.e., with≥2 visits with cognitive composites, self-reported health history, and concurrent medication records) were included in the analysis. Exclusionary criteria (*n* = 567) included: only completed 1 visit (*n* = 221), no concurrent medication records (n = 1), no LIBRA index (*n* = 330), non-progressive cognitive due to longstanding conditions (e.g., learning disability, *n* = 10), or missing cognition status (*n* = 5). Based on these criteria, our dataset included baseline and available follow-up visits from 1039 participants.

### Study Methods

#### Number of Medications and Co-Morbidity Tally

Medications at each visit were quantified by a combination of self-report itemization, medical record, and by bringing their actual medications to the study visit. Medications were categorized by research staff into prescription and over-the-counter (OTC) medicines. The total number of prescriptions and OTC drugs were counted for each participant at each visit, up to 15 per type. The co-morbidity tally was defined at each study visit as self-reported chronic conditions (e.g., heart disease, hypertension, diabetes, liver disorder; Supplementary Table S1)

For analyses, we converted the number of prescriptions and co-morbidities to z-scores. In addition, we applied published cut-offs to divide the participants into three groups: no Polypharmacy (0-4 medications), Polypharmacy (5-9 medications), and Hyper-Polypharmacy (≥ 10 medications) (O’Dwyer et al., 2016; Masnoon et al., 2017).

#### Cognitive Composite Measures

At each visit, participants completed a comprehensive neuropsychological battery (details in Johnson et al., 2018). For these analyses, we examined a three-test cognitive composite consisting of the Rey Auditory Verbal Learning Test total score, delayed recall from Logical Memory (LM-II; WIMS-R), and Digit Symbol (WAIS-R) (see Jonaitis et al., 2019). This composite was designed to resemble the PACC described by Donohue et al. (2014), omitting Mini-Mental State Exam (MMSE) due to its limited sensitivity in middle-aged healthy samples (Donohue et al., 2014; Mormino et al., 2017; Jonaitis et al., 2019). Four domain-specific cognitive composites were also examined including working memory (Koscik et al., 2014), executive function (EF), immediate learning, and delayed recall (Clark et al., 2016). The tests contributing to each composite are shown in Table 1.

**TABLE 1:** Mapping of tests (rows) to five cognitive composites (columns).

Raw scores	Working memory	Immediate learning	Delayed recall	Executive function	PACC3
Rey AVLT Total	—	X	—	—	X
Rey AVLT Delayed	—	—	X	—	—
WMS-R Logical Memory-I	—	X	—	—	—
WMS-R Logical Memory-II	—	—	X	—	X
BVMT-R Total	—	X	—	—	—
BVMT-R Delayed	—	—	X	—	—
Stroop Color-Word	—	—	—	X	—
TMT Part A	—	—	—	—	—
TMT Part B	—	—	—	X	—
WAIS-R Digit Symbol	—	—	—	X	X
Digit span Forward	X	—	—	—	—
Digit span Backward	X	—	—	—	—
Letter-Number Sequence	X	—	—	—	—

Abbreviations: PACC-3, Preclinical Alzheimer Cognitive Composite (3 tests); AVLT (Schmidt, 1996), Auditory-Verbal Learning Test; BVMT-R (Benedict, 1997), Brief Visuospatial Memory Test-Revised; Stroop Color-Word (Trenerry et al., 1989), Stroop test, Color-Word Interference; TMT (Reitan, 1958), Trail Making Test; WMS-R (Wechsler, 1987), Wechsler Memory Scale-Revised; WAIS-R Digit Symbol (Wechsler, 1997), Digit Symbol subtest of the Wechsler Adult Intelligence Scale-Revised.

Note. X in a cell indicates that the test represented in that row contributed to that column’s composite.

#### Health Measures and Covariates

Self-rated current health (SRH) represented a measure of health that was distinct from objective measures of clinical, functional, and psychosocial status (Walker et al., 2004). SRH was measured using a 5-point scale (1 = poor, 2 = fair, 3 = good, 4 = very good, 5 = excellent) in response to the question, “How would you rate your current health?”.

The LIfestyle for BRAin health (LIBRA) index incorporated 11 modifiable risk and protective factors for dementia including low/moderate alcohol consumption; the presence of cardiovascular disease, physical inactivity, renal dysfunction, diabetes, high cholesterol, smoking, obesity, hypertension, depression, and high cognitive activity (Vos et al., 2017). The risk scores were calculated using previously established relative risks from large epidemiological studies (Deckers et al., 2015) with higher scores indicating a higher lifestyle-related risk of dementia.

The 20-item Center for Epidemiologic Studies-Depression scale (CES-D) (Radloff, 1977) was completed by each participant as part of the health history form.

Other variables of interest included age, sex, education, race, participant site (Madison, La Crosse, and Milwaukee, baseline Wide Range Achievement Test– 3 (WRAT-3) Reading subtest standard score (Wilkinson, 1993), and practice (the number of prior exposures to the battery). *APOE* ε2/ε3/ε4 and 20 common genetic variants from the International Genomics of Alzheimer’s Project consortium were genotyped using competitive allele-specific polymerase chain reaction-based genotyping assays (LGC Genomics, Beverly, MA) as described previously (Johnson et al., 2018). In these analyses, *APOE* genotype was expressed as a binary categorical variable, with participants classified as carriers (one or more ε4 alleles present) or non-carriers (no ε4 allele present).

### Statistical Methods

Our analyses focus on composite scores rather than individual test scores for two primary reasons: 1) use of composites reduces the overall number of tests thereby reducing the probability of spurious or irreproducible findings (Gelman and Geurts, 2017); further, recent analyses in our data (Jonaitis et al., 2019) demonstrated that composites generally have less within-person variability and greater sensitivity to AD biomarker-related decline.

Statistical analyses for Aim 1: Baseline sample characteristics (e.g., demographics, cognitive composites, health measures) were compared regarding sex and polypharmacy status groups using tests appropriate to the distribution and number of groups being compared. Chi-square or Fisher’s exact was performed for categorical comparisons of 2 groups; t-test or analysis of variance (ANOVA) was performed for normally distributed variables; Mann-Whitney U test was used for comparing 2 groups with non-normal distributed variables; Kruskal-Wallis was used for comparing 3 groups with non-normal data.

Statistical analyses for Aim 2: To characterize the associations between comorbidities, medication numbers, SRH, and objective measures of health (including LIBRA and depression), between-outcome estimates were calculated using the repeated measures correlation (rmcorr), which adjusts for between-subjects performance differences (Bakdash and Marusich, 2017). Because repeated measures correlation takes into account non-independence (within-person), it tends to yield much greater power than data that are averaged to meet the independent and identically distributed assumption for simple regression/correlation. Confidence intervals were computed using bootstrap by resampling 100 every time as recommended by Cumming (2014).

Statistical analyses for Aim 3: We used linear mixed-effects models and a set of models to examine associations between key predictors (e.g., medications, SRH) and cognitive trajectories (random intercept and age-related slope; unstructured covariance). As noted previously, the number of medications and co-morbidities were standardized to a mean of 0 and a standard deviation of 1 (z-scores). We centered age at 60 and replaced the continuous measure of years of education with discrete categories (Education Years≥16, College = “Y”; Education Years <16, College = N).

To control Type I error relating to domain differences in the impact of key predictors, we first assessed the effect of these predictors on cognition overall by constructing a single mixed-effects model encompassing all cognitive data. In these models, the cognitive test domain (PACC3, executive function, working memory, immediate learning, and delayed recall) and its interactions with medications, comorbidities, and SRH were included as predictors of cognition. After determining that associations between these predictors and cognitive performance differed across the test domains, fits of model 1 (base model) (covariates of centered age, sex, race, site, WRAT-III Reading, education level, *APOE* ε4 carrier status, and practice effects) for each composite were compared with model 2 that included the number of prescriptions, comorbidities, and SRH. Only significant interactions were retained in the final model outputs reported. Collinearity and influence diagnostics were examined for final models. We report Akaike information criterion (AIC)/Bayesian information criterion (BIC) and Likelihood ratio tests and interpret the models with the highest order significant predictors of interest. For significant interactions, simple slope analyses were conducted to interpret the significant interactive effects (Aiken et al., 1991; Cohen et al., 2013).

We included the following secondary analyses. First, to understand more about the association between self-reported medications and comorbidities, we extended aim 2 and examined whether the number of medications and comorbidities interacted with each other or modified age trajectories. Exploratory analyses related to Aim 1 included examining whether there was evidence that the polypharmacy group was associated with cognitive status at the last visit (cognitively unimpaired vs clinically impaired). Exploratory analysis for Aim 1 and 3 to identify potential moderating effects of sex in the association between polypharmacy, comorbidities, and cognitive performance, we examined whether there was a two-way interaction that varies across levels of a third variable for each cognitive composite domain, three-way interactions were examined (numbers of medications, co-morbidities, and centered age; sex, numbers of medications and centered age; sex, co-morbidities, and centered age; sex, SRH and centered age). Only significant interactions were retained in the final model outputs reported.

The results are reported in accordance with the Strengthening the Reporting of Observational Studies in Epidemiology (STROBE) guidelines for reporting observational studies (von Elm et al., 2014). Diagnostic checks were performed on all estimated regression. The results were considered statistically significant with a significance level of ≤ 0.05 (unadjusted for multiple comparisons). All analyses were performed in R version 4.0.0.

## Results

### Demographic and Baseline Characteristics

1039 participants had at least two cognitive composites, self-reported health history concurrent medication records, and thus eligible for inclusion in the analysis. The mean age of this sample was 59.0 years (SD 6.6) at the indexed baseline visit; details on demographic characteristics are found in Table 2 overall and by sex. Participants could report up to 31 co-morbidities; the highest sum of co-morbidities was 17 (Supplementary Table S1). The highest sum of prescription medications was 15. Of note, female participants took more prescriptions and OTCs than men, but men and women did not differ significantly in terms of total co-morbidities (Table 2). Male participants scored lower on immediate learning, delayed recall, executive function, and PACC3. Female participants had a lower LIBRA index on average compared to male participants.

**TABLE 2:** Demographics and baseline characteristics of participants by sex.

	Overall	Male	Female	p^b^
N	1039	325 (%)	714 (%)	
Baseline age^a^ [mean (sd)]	59.0 (6.6)	59.4 (6.6)	58.8 (6.6)	0.14
Age at last visit [mean (sd)]	65.6 (6.7)	66.2 (6.8)	65.3 (6.7)	0.06
Years of follow-up (median [Q1–Q3])	6.8 [4.4–9.1]	6.9 [4.3–9.4]	6.8 [4.5–8.9]	0.24
Number of Visits (median [Q1–Q3])	4.0 [3.0–5.0]	4.0 [3.0–5.0]	4.0 [3.0–4.0]	0.21
Race (*n* (%))				0.34
American Indian/Native American	5 (0.5)	1 (0.3)	4 (0.6)	
Asian	2 (0.2)	2 (0.6)	0 (0.0)	
Black/African American	26 (2.5)	7 (2.2)	19 (2.7)	
White/Caucasian	994 (95.9)	311 (95.7)	683 (95.9)	
Spanish/Hispanic	9 (0.9)	4 (1.2)	5 (0.7)	
Education Years (median [Q1–Q3])	16.0 [14.0–18.0]	16.0 [14.0–18.0]	16.0 [14.0–18.0]	0.02
*APOE* ε4 carriers [*n* (%)]	397 (38.2)	281 (39.4)	116 (35.7)	0.29
Site (*n* (%))				0.29
LaCrosse	243 (23.4)	67 (20.6)	176 (24.6)	
MKE	53 (5.1)	15 (4.6)	38 (5.3)	
MSN	743 (71.5)	243 (74.8)	500 (70.0)	
WRAT-III Reading [mean (sd)]	108.2 (8.5)	107.3 (9.2)	108.5 (8.2)	0.07
CES-D score (median [Q1–Q3])	5.0 [2.0–9.0]	4.0 [2.0–9.0]	5.0 [2.0–9.0]	0.03
Num_prescriptions (median [Q1–Q3])	2.0 [0.0–4.0]	1.0 [0.0–3.0]	2.0 [1.0–4.0]	0.002
Num_prescriptions group (%)				0.02
NP (0–4)	858 (82.6)	278 (85.5)	580 (81.2)	
PP (5–9)	165 (15.9)	39 (12.0)	126 (17.6)	
HP (≥10)	16 (1.5)	8 (2.5)	8 (1.1)	
Num_OTC (median [Q1–Q3])	2.0 [1.0–4.0]	2.0 [0.0–4.0]	2.0 [1.0–4.0]	<0.001
Co-morbidities (median [Q1–Q3])	3.0 [2.0–4.0]	3.0 [1.0–4.0]	3.0 [2.0–5.0]	0.04
LIBRA index [mean (sd)]	0.9 (2.2)	1.1 (2.1)	0.8 (2.3)	0.04
SRH(%)				0.81
Poor	2 (0.2)	1 (0.3)	1 (0.1)	
Fair	55 (5.3)	20 (6.2)	35 (4.9)	
Good	358 (34.7)	108 (33.5)	250 (35.2)	
Very good	447 (43.3)	139 (43.2)	308 (43.4)	
Excellent	170 (16.5)	54 (16.8)	116 (16.3)	
Working memory z scale [mean (sd)]	0.11 (0.97)	0.15 (1.02)	0.10 (0.95)	0.47
Immediate learning [mean (sd)]	0.07 (0.77)	−0.21 (0.81)	0.19 (0.71)	<0.001
Delayed recall [mean (sd)]	0.06 (0.75)	−0.19 (0.81)	0.18 (0.70)	<0.001
Executive function [mean (sd)]	0.05 (0.80)	−0.20 (0.84)	0.16 (0.75)	<0.001
PACC3 [mean (sd)]	0.06 (0.74)	−0.28 (0.76)	0.21 (0.68)	<0.001

a Baseline age refers to the age at which the cognitive composites were available for all 5 composites (typically visit 2). Abbreviations: APOE, apolipoprotein E; MKE, Milwaukee; MSN, Madison; WRAT3, wide range achievement test (third edition), CES-D, center for epidemiologic studies depression scale; Num_prescription, number of prescription medications; NP, No polypharmacy; PP, polypharmacy; HP, hyper polypharmacy; Num_OTC, number of over-the-counter medications; LIBRA, lifestyle for BRAin, health; SRH, Self rated health; PACC-3, preclinical alzheimer cognitive composite (3 tests), Q1–Q3, first to third quantile; SD, standard deviation.

b Statistical tests: chi-square or Fisher’s exact for categorical; between-sample t-tests for continuous where mean (SD) reported; Mann-Whitney U Test for continuous where median [Q1–Q3] reported.

Table 3 presents baseline characteristics by polypharmacy group. Average age (sd) in each baseline polypharmacy group increased with numbers of medications: 58.4 (6.6) no polypharmacy (NP) (<5 prescription medications); 61.6 (5.6) polypharmacy (PP) (5-9); and 62.7 (6.8) hyper-polypharmacy (HP) (≥ 10). Polypharmacy groups did not differ in WRAT3 reading, years of education, race, or *APOE*4 carriage, but differed on sex and study site; follow-up pairwise comparisons showed the polypharmacy group had a higher proportion of women than the other two groups. Polypharmacy groups also differed on depression, number of OTCs, co-morbidities, LiBRA indices, and SRH, with worse values associated with more medications. The polypharmacy groups differed on baseline executive function after adjustment for age, WRAT reading, and sex; pairwise comparisons showed lower EF in the polypharmacy group compared to the no polypharmacy group. The *n* (%) cognitively impaired at the last cognitive visit was 19 (2.4), 14 (6.2), and 2 (6.7) for the NP, PP, and HP groups, respectively (*p* = 0.014).

**TABLE 3:** Demographics participants by prescription medication subgroups.

	NP	PP	HP	P value^b^	Difference pairs
N	858 (82.6%)	165 (15.9%)	16 (1.5%)		
Baseline age^a^ [mean (sd)]	58.4 (6.6)	61.6 (5.6)	62.7 (6.8)	<0.001	NP versus PP, HP
Years of follow-up (median [Q1–Q3])	6.9 [4.5–9.2]	6.4 [3.3–8.5]	3.8 [2.3–6.2]	<0.001	All pairs
Female (*n* (%))	580 (67.6)	126 (76.4)	8 (50.0)	0.02	PP versus NP, HP
Number of Visits (median [Q1–Q3])	4.0 [3.0–5.0]	4.0 [2.0–4.0]	3.0 [2.0–3.3]	0.002	All pairs
Race [*n* (%)]				0.24	
American Indian/Native American	4 (0.5)	1 (0.6)	0 (0.0)		
Asian	2 (0.2)	0 (0.0)	0 (0.0)		
Black/African American	17 (2.0)	7 (4.2)	2 (12.5)		
White/Caucasian	824 (96.3)	156 (94.5)	14 (87.5)		
Spanish/Hispanic	8 (0.9)	1 (0.6)	0 (0.0)		
Education Years (median [Q1–Q3])	16.0 [14.0–18.0]	16.0 [14.0–18.0]	17.0 [14.0–17.3]	0.58	
*APOE* ε4 carriers [*n* (%)]	325 (37.9)	67 (40.6)	5 (31.2)	0.71	
Site (n (%))				<0.001	HP versus NP, PP
LaCrosse	197 (23.0)	40 (24.2)	6 (37.5)		
MKE	33 (3.8)	16 (9.7)	4 (25.0)		
MSN	628 (73.2)	109 (66.1)	6 (37.5)		
WRAT-III Reading [mean (sd)]	108.0 (8.7)	109.3 (7.4)	108.4 (7.4)	0.23	
CES-D score (median [Q1–Q3])	4.0 [2.0–8.0]	5.0 [2.0–10.0]	9.0 [6.8–15.5]	0.001	HP versus NP, PP
Num_OTC (median [Q1–Q3])	2.0 [1.0–4.0]	3.0 [2.0–5.0]	2.0 [0.8–4.0]	<0.001	NP versus PP
Co-morbidities (median [Q1–Q3])	3.0 [1.0–4.0]	4.0 [3.0–6.0]	8.0 [5.5–10.0]	<0.001	All pairs
LIBRA index [mean (sd)]	0.7 (2.1)	1.6 (2.4)	3.1 (2.5)	<0.001	All pairs
SRH(%)				<0.001	All pairs
Poor	1 (0.1)	1 (0.6)	0 (0.0)		
Fair	31 (3.6)	16 (9.7)	8 (50.0)		
Good	271 (31.8)	79 (47.9)	8 (50.0)		
Very good	389 (45.7)	58 (35.2)	0 (0.0)		
Excellent	159 (18.7)	11 (6.7)	0 (0.0)		
Working memory [mean (sd)]	0.14 (0.96)	−0.03 (0.98)	−0.08 (1.14)	0.11	
Immediate learning^c^ [lsmean (se)]	0.01 (0.03)	−0.05 (0.06)	−0.02 (0.24)	0.63	
Delayed recall^c^ [lsmean (se)]	0.02 (0.03)	−0.02 (0.06)	−0.23 (0.24)	0.51	
Executive function^c^ [lsmean (se)]	0.06 (0.03)	−0.13 (0.06)	−0.19 (0.24)	0.01	NP versus PP
PACC3^c^ [lsmean (se)]	0.02 (0.02)	−0.09 (0.06)	−0.31 (0.22)	0.08	
Cognitive impairment at last visit [*n* (%)]	19 (2.4)	14 (6.2)	2 (6.7)	0.014	NP versus PP

a Baseline age refers to age at which the cognitive composites were available for all 5 composites (typically visit 2).

b Statistical tests: analysis of variance (ANOVA) for continuous where mean (SD) reported; Kruskal-Wallis for continuous where median [Q1–Q3] reported. Post hoc pairwise group differences at unadjusted *p* < 0.05 noted in right-hand column. For example, NP versus PP, HP indicates group NP differed from group PP and group HP in separate pairwise comparisons.

c Adjusting for age, sex, and WRAT-III Reading, since the cognitive composite scores differ by sex in Table 2.

### Associations Between Comorbidities, Polypharmacy, Self-Reported and Objective Measures of Health (including SRH, LIBRA, and Depression)

**FIGURE 1:**
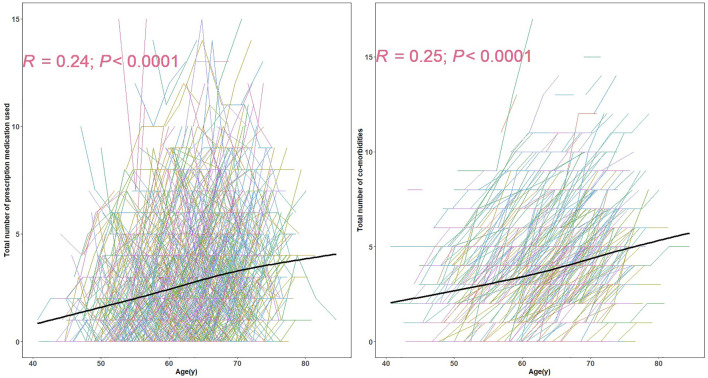
The spaghetti plot of prescription medication used and the number of co-morbidities.Each line represents the longitudinal pattern of number of prescription medications **(left)** or number of self-reported co-morbidities at different ages **(right)**. The black line in each represents the estimated average numbers at different ages. R is the spearman correlation coefficient.

The prevalence of polypharmacy and hyper-polypharmacy increased from 17.4% (181/1039) at baseline to 24.7% (257/1039) at the last visit in this relatively healthy cohort. The numbers of prescription medications and co-morbidities were positively correlated with age (Figure 1). The mean number of prescriptions (corresponding z-scores) for 45–55, 55–65, and 65–75 year olds were 1.76 (−0.34), 2.44 (−0.07) and 3.23 (0.24), separately. The mean number of comorbidities (corresponding z-scores) for 45–55, 55–65, and 65–75 year olds were 2.81 (0.07), 3.45 (0.32) and 4.25 (0.63), separately.

**FIGURE 2:**
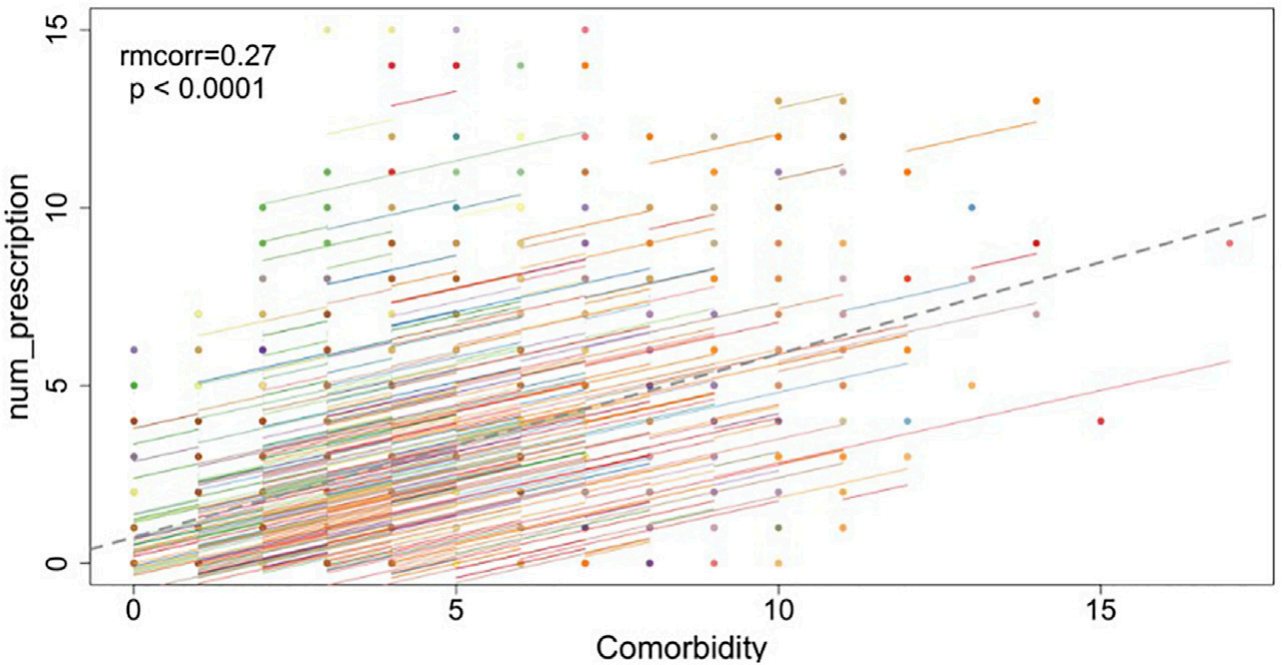
The repeated measures correlation between co-morbidities and the number of prescription medications. This figure depicts the repeated measures correlation between co-morbidities and the number of prescription medications. Observations from the same participant are given the same color, with corresponding lines to show the repeated measures correlation (rmcorr) fit for each participant. The gray dotted line is the regression line between co-morbidities and the number of prescription medications, ignoring the participant variable.

The number of prescription medications was, not surprisingly, positively associated with the self-reported number of co-morbidities ( $r_{m}=0.27$ , 95% CI [0.23, 0.30], p-value < 0.0001; Figure 2).

**FIGURE 3:**
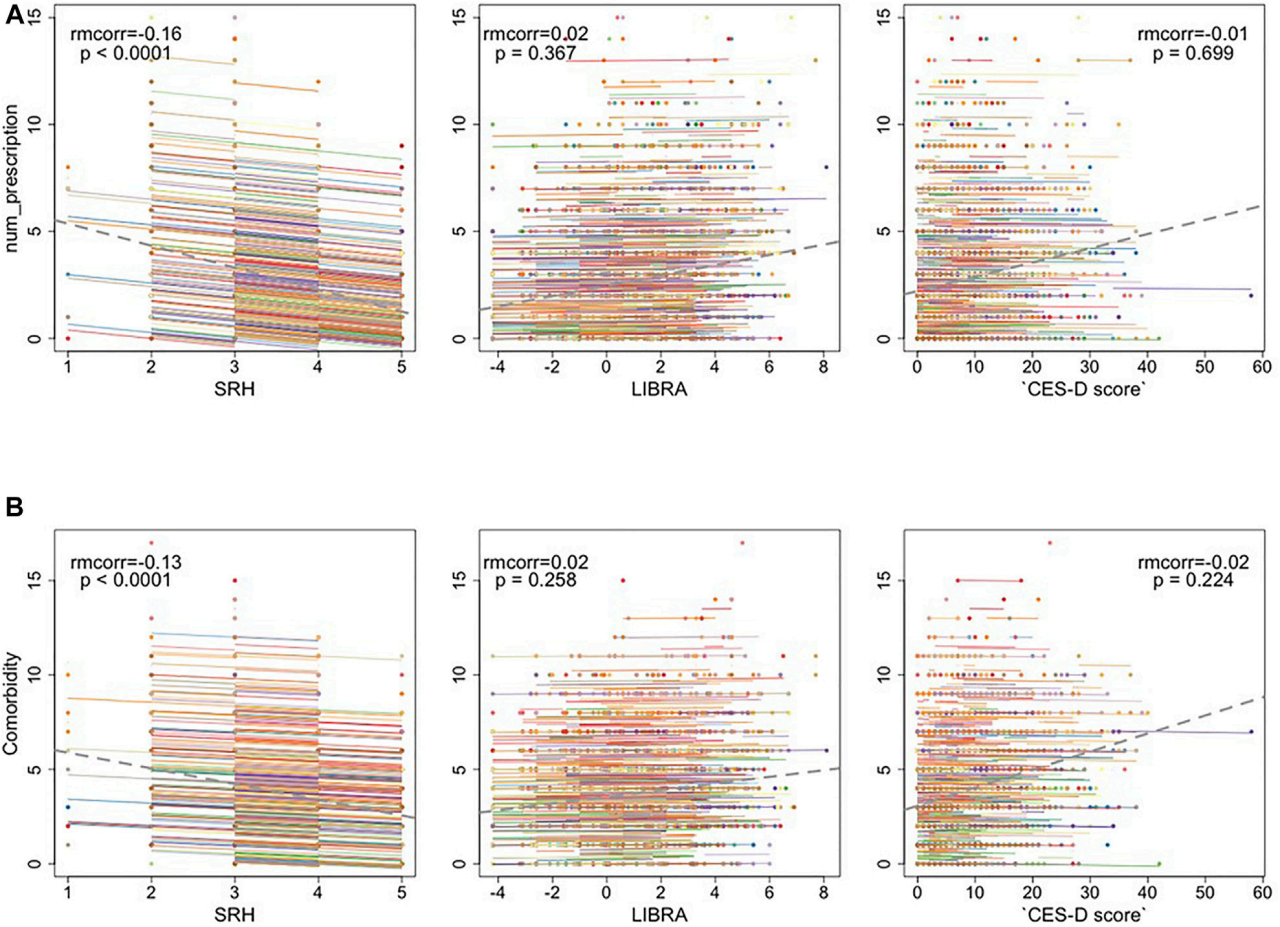
**(A)** The repeated measures correlation between the number of prescription medications and Self-rated health (SRH), LIfestyle for BRAin Health Index (LIBRA), and CES-D depression scores **(B)** The repeated measures correlation between the number of co-morbidities and SRH, LIBRA, and depression. The dotted gray line is the regression line between x and y, ignoring the participant variable.

Poorer SRH was associated with more prescriptions and comorbidities (Figure 3). And this correlation is not simply induced by between-person variation but holds within participants. Associations were not significant for these pairs: number of prescription medications and LIBRA, depression (Figure 3A); number of comorbidities and LIBRA, depression (Figure 3B).

### Associations Between Polypharmacy, Co-Morbidities, and Longitudinal Cognitive Performance

Preliminary multivariate analyses indicated that key predictors behaved differently across cognitive outcomes. Thus, model sets were run separately for each cognitive composite. All regressions passed the regression diagnostics. For each outcome, follow-up simple slopes analyses are reported for significant predictor*centered age interactions for low *vs* high values of the predictor (e.g., 10th vs 90th percentile).

Table 4 summarizes the results of Models 1-3 for PACC3. Model 1 is with covariates only, Model 2 adds health predictors of interest, and Model 3 adds the interactions with age. Younger participants, females, those with higher WRAT-3 reading scores, those with a college degree, and those not carrying APOE4 performed better on the PACC3. Model 2 and Model 3 retained a significant age* prescriptions interaction, had improved AIC over Model 1 as well as a significant log-likelihood test. Slightly less annual decline in PACC3 was observed in the context of 10th percentile prescriptions z-score (Δ_PACC3_ = −0.05 SD/yr) compared to 90th percentile prescriptions z-score (Δ_PACC3_ = −0.07 SD/yr) (Supplementary Figure S1).

**TABLE 4:** The associations between medications, co-morbidities, and PACC3 z-scores.

Predictors	Model 1^c^		Model 2^c^		Model 3^c^	
	β (95%CI)	p	β (95%CI)	p	β (95%CI)	p
Center_age^a^	−0.06 (−0.07–−0.05)	**<0.001**	−0.06 (−0.07–−0.05)	**<0.001**	−0.06 (−0.07–−0.05)	**<0.001**
Female	0.63 (0.52–0.74)	**<0.001**	0.64 (0.53–0.75)	**<0.001**	0.64 (0.53–0.75)	**<0.001**
White	0.09 (−0.26–0.44)	0.601	0.09 (-0.26–0.44)	0.607	0.09 (−0.26–0.44)	0.597
LaCrosse	−0.05 (−0.17–0.08)	0.444	−0.05 (−0.17–0.07)	0.423	−0.05 (−0.17–0.08)	0.47
MKE	−0.45 (−0.79–−0.11)	**0.009**	−0.41 (−0.75–−0.07)	**0.018**	−0.42 (−0.76–−0.08)	**0.016**
WRAT-III Reading	0.03 (0.02–0.03)	**<0.001**	0.03 (0.02–0.03)	**<0.001**	0.03 (0.02–0.03)	**<0.001**
College	0.28 (0.16–0.40)	**<0.001**	0.28 (0.16–0.40)	**<0.001**	0.28 (0.16–0.40)	**<0.001**
*APOE* ε4 carriers	−0.12 (−0.23–−0.02)	**0.02**	−0.12 (−0.22–−0.01)	**0.027**	−0.12 (−0.22–−0.01)	**0.025**
Practice	0.12 (0.09–0.14)	**<0.001**	0.12 (0.10–0.15)	**<0.001**	0.12 (0.10–0.15)	**<0.001**
SRH			0.02 (−0.01–0.05)	0.251	0.02 (−0.01–0.05)	0.226
z-comorbidities			−0.05 (−0.10–−0.01)	**0.024**	−0.05 (−0.10–−0.01)	**0.023**
z-num_prescriptions			−0.03 (−0.07–0.00)	0.066	−0.01 (−0.05–0.03)	0.573
Center_age^a^ z- num_prescriptions					−0.01 (−0.01–−0.00)	**0.001**
BIC/AIC	5652.2/5567.2		5661.5/5558.4		5659.0/5549.8	
LR test^b^				**0.002**		**<0.001**
Marginal/Conditional R^2^	0.301/0.850	0.306/0.849	0.308/0.850			

The bold values indicate the results are statistically significant (p < 0.05).

a Center_age = center age at visit on 60; MKE, Milwaukee; MSN, Madison; WRAT3, wide range achievement test (third edition); College, education years ≥ 16, *APOE*, apolipoprotein E; SRH, self rated health; z-comorbidities, z-scores of number of co-morbidities; z-num_prescriptions, z-scores of number of prescriptions; BIC = bayesian information criterion; AIC, akaike information criterion; LR, likelihood ratio. Male; Madison, Non-white and No college are reference levels. p-value (< 0.05) indicates that you can reject the null hypothesis that the coefficient is equal to 0.

b Test for the model 1 vs model 2, model 1 vs model 3. p-value (< 0.05) means adding the variables together explained a significant amount of variability in the outcome.

c Model 1: covariates + random effects; Model 2: Model 1 + SRH, z-num_prescriptions, z-comorbiditites; Model 3: Model 2 + significant interactions between age and z-num_prescriptions.

Table 5 summarizes the results of Models 1-3 for EF. Younger participants, females, those with higher WRAT-3 reading scores performed better on EF. Model fits statistics indicated that model 2 and model 3 accounted for significantly more variability than Model 1. The interaction between number of comorbidities and age was retained in model 3. Comparison of the simple age-related EF slopes suggested the high co-morbidities z-score group declined 0.01 SD/year faster than the low co-morbidities. (Supplementary Figure S1).

**TABLE 5:** The associations between medications, co-morbidities and EF z-scores.

Predictors	Model 1^b^	Model 2^b^			Model 3^b^	
	β (95%CI)	p	β (95%CI)	p	β (95%CI)	p
Center_age^a^	−0.07 (−0.08 – −0.06)	**<0.001**	−0.07 (−0.07–−0.06)	**<0.001**	−0.07 (−0.08–−0.06)	**<0.001**
Female	0.38 (0.27–0.50)	**<0.001**	0.40 (0.29–0.52)	**<0.001**	0.40 (0.28–0.51)	**<0.001**
White	0.12 (−0.24–0.48)	0.519	0.12 (−0.24–0.48)	0.524	0.12 (-0.24–0.48)	0.511
LaCrosse	−0.06 (−0.19–0.07)	0.364	−0.06 (−0.19–0.06)	0.334	−0.06 (−0.18–0.07)	0.362
MKE	−0.46 (−0.81–−0.11)	**0.011**	−0.40 (−0.74–−0.05)	**0.026**	-0.40 (-0.75–−0.05)	**0.024**
WRAT-III Reading	0.02 (0.01–0.03)	**<0.001**	0.02 (0.01–0.03)	**<0.001**	0.02 (0.01–0.03)	**<0.001**
College	0.12 (0.00–0.25)	**0.049**	0.12 (-0.00–0.24)	0.052	0.12 (-0.00–0.24)	0.052
*APOE* ε4 carriers	−0.08 (−0.19–0.02)	0.128	−0.07 (−0.18–0.03)	0.171	-0.08 (-0.18–0.03)	0.156
Practice	0.08 (0.06–0.11)	**<0.001**	0.09 (0.07–0.11)	**<0.001**	0.09 (0.07–0.12)	**<0.001**
SRH			0.02 (−0.01–0.05)	0.165	0.02 (-0.01–0.05)	0.156
z-comorbidities			−0.06 (−0.11–−0.02)	**0.005**	−0.04 (−0.09–0.00)	0.076
z-num_prescriptions			−0.06 (−0.09–−0.03)	**<0.001**	−0.06 (−0.09–−0.03)	**<0.001**
Center_age^a^ z-comorbiditites					−0.01 (−0.01–−-0.00)	**0.002**
BIC/AIC	4834.2/4749.5		4825.5/4722.7		4823.8/4714.9	
LR test^c^				**<0.001**		**<0.001**
Marginal/Conditional R^2^	0.257/0.890		0.269/0.890		0.274/0.890	

The bold values indicate the results are statistically significant (p < 0.05).

a Center_age, center age at visit on 60; MKE, Milwaukee; MSN, Madison; WRAT3, wide range achievement test (third edition), College, education years ≥ 16, *APOE*, apolipoprotein E; SRH, self rated health; z-comorbidities, z-scores of number of co-morbidities; z-num_prescriptions, z-scores of number of prescriptions; BIC, bayesian information criterion; AIC, akaike information criterion; LR, likelihood ratio. Male, Madison, Non-white and No college are reference levels. p-value (< 0.05) indicates that you can reject the null hypothesis that the coefficient is equal to 0.

b Model 1: covariates + random effects; Model 2: Model 1 + SRH, z-num_prescriptions, z-comorbiditites; Model 3: Model 2 + significant interactions between age and z-comorbiditites.

c Test for the model 1 vs model 2, model 1 vs model 3. p-value (< 0.05) means adding the variables together explained a significant amount of variability in the outcome.

Table 6 summarizes the results of Models 1-3 for working memory. Main effects of age, sex, WRAT-3 reading, site, APOE4 carriage, and practice were significant, such that younger participants, males, those with higher WRAT-3 reading scores, and those not carrying *APOE4* performed better on working memory. Model 3, retained a significant age*comorbidities interaction and had improved an AIC over Model 1 as well as a significant log-likelihood test. A slightly less annual decline in working memory was observed in the context of 10th percentile comorbidities (Δ_WM_ = −0.025 SD/yr) compared to 90th percentile comorbidities (Δ_WM_ = −0.04 SD/yr) (Supplementary Figure S1).

**TABLE 6:** The associations between medications, co-morbidities and working memory z-scores.

Predictors	Model 1^b^	Model 2^b^			Model 3^b^	
	β (95%CI)	p	β (95%CI)	p	β (95%CI)	p
Center_age^a^	−0.03 (−0.04–−0.02)	**<0.001**	−0.03 (−0.04–−0.02)	**<0.001**	−0.03 (−0.04–−0.02)	**<0.001**
Female	−0.13 (−0.24–−0.01)	**0.032**	−0.13 (−0.24–−0.01)	**0.033**	−0.13 (−0.25–−0.02)	**0.026**
White	0.01 (−0.36–0.38)	0.952	0.01 (−0.36–0.38)	0.965	0.02 (−0.35–0.38)	0.929
LaCrosse	0.01 (−0.12–0.14)	0.895	0.01 (−0.12–0.14)	0.911	0.01 (−0.12–0.14)	0.911
MKE	−0.37 (−0.73–−0.02)	**0.038**	−0.37 (−0.72–−0.01)	**0.042**	−0.38 (−0.73 – -0.02)	**0.037**
WRAT-III Reading	0.04 (0.04–0.05)	**<0.001**	0.04 (0.04–0.05)	**<0.001**	0.04 (0.04–0.05)	**<0.001**
College	0.03 (−0.10–0.15)	0.693	0.02 (−0.10–0.15)	0.723	0.03 (−0.10–0.15)	0.688
*APOE* ε4 carriers	−0.17 (−0.28–−0.06)	**0.003**	−0.17 (−0.28–−0.06)	**0.003**	-0.17 (-0.28 – -0.06)	**0.003**
Practice	0.07 (0.04–0.09)	**<0.001**	0.06 (0.04–0.09)	**<0.001**	0.06 (0.04–0.09)	**<0.001**
SRH			−0.01 (−0.05–0.02)	0.54	−0.01 (−0.05–0.02)	0.558
z-comorbidities			0.03 (−0.02–0.07)	0.254	0.06 (0.01–0.11)	**0.025**
z-num_prescriptions			−0.03 (−0.06–0.01)	0.143	−0.03 (−0.06–0.01)	0.131
Center_age^a^ z-comorbiditites					−0.01 (−0.01–−0.00)	**0.002**
BIC/AIC	5996.3/5923.5		6017.5/5926.4		6015.7/5918.5	
LR test^c^				0.384		**0.012**
Marginal/Conditional R^2^	0.164/0.791		0.165/0.791		0.168/0.792	

The bold values indicate the results are statistically significant (p < 0.05).

a Center_age, center age at visit on 60; MKE, Milwaukee; MSN, Madison; WRAT3, wide range achievement test (third edition); College, education years ≥ 16, *APOE*, apolipoprotein E; SRH, self rated health; z-comorbidities, z-scores of number of co-morbidities; z-num_prescriptions, z-scores of number of prescriptions; BIC, bayesian information criterion; AIC, akaike information criterion; LR, likelihood ratio. Male, Madison, Non-white and No college are reference levels. p-value (< 0.05) indicates that you can reject the null hypothesis that the coefficient is equal to 0.

b Model 1: covariates + random effects; Model 2: Model 1 + SRH, z-num_prescriptions, z-comorbiditites; Model 3: Model 2 + significant interaction between age and z-comorbiditites.

c Test for the model 1 vs model 2, model 1 vs model 3. p-value (< 0.05) means adding the variables together explained a significant amount of variability in the outcome.

Table 7 summarizes the results of Models 1-3 for immediate learning. Younger participants, females, higher WRAT-3 reading scores, having a college degree or no APOE4 were significantly associated with higher immediate learning. Model fits statistics indicated that model 3 but not model 2 accounted for significantly more variability than model 1. Both numbers of prescriptions*age and SRH*age were retained in model 3. Two interactions (centered age * number of prescriptions and centered age * SRH) were retained in model 3. Comparison of the simple age-related immediate learning suggested the high prescription z-score group declined 0.01 SD/year faster than the low prescription group. Comparison of the simple age-related SRH relationship suggested the poor SRH group declined 0.03 SD/year faster than the excellent SRH group (Supplementary Figure S1).

**TABLE 7:** The associations between medications, co-morbidities and immediate learning z-scores.

Predictors	Model 1^b^	Model 2^b^			Model 3^b^	
	β (95%CI)	p	β (95%CI)	p	β (95%CI)	p
Center_age^a^	−0.05 (−0.06–−04)	**<0.001**	−0.05 (−0.06–−0.04)	**<0.001**	−0.08 (−0.10–−0.06)	**<0.001**
Female	0.50 (0.39–0.62)	**<0.001**	0.51 (0.39–0.62)	**<0.001**	0.50 (0.39–0.61)	**<0.001**
White	0.22 (−0.13–0.58)	0.223	0.22 (−0.13–0.58)	0.222	0.23 (−0.13–0.59)	0.209
LaCrosse	0.05 (−0.07–0.18)	0.414	0.05 (−0.07–0.18)	0.416	0.06 (−0.07–0.19)	0.356
MKE	−0.25 (−0.59–0.10)	0.16	−0.24 (−0.59–0.10)	0.169	−0.25 (-0.59–0.10)	0.164
WRAT-III Reading	0.03 (0.02–0.04)	**<0.001**	0.03 (0.02–0.04)	**<0.001**	0.03 (0.02–0.04)	**<0.001**
College	0.25 (0.13–0.38)	**<0.001**	0.26 (0.13–0.38)	**<0.001**	0.26 (0.13–0.38)	**<0.001**
*APOE* ε4 carriers	−0.12 (−0.23–−0.01)	**0.027**	−0.12 (−0.23–−0.01)	**0.029**	−0.12 (−0.22–−0.01)	**0.031**
Practice	0.18 (0.15–0.20)	**<0.001**	0.18 (0.15–0.20)	**<0.001**	0.18 (0.15–0.20)	**<0.001**
SRH			0.004 (−0.03–0.04)	0.844	−0.005 (−0.04–0.03)	0.816
z-comorbidities			−0.01 (−0.06–0.04)	0.6	−0.01 (−0.06–0.04)	0.666
z-num_prescriptions			0.001 (−0.04–0.04)	0.966	0.02 (−0.02–0.06)	0.367
Center_age^a^ z- num_prescriptions					−0.005 (−0.01–−0.001)	**0.029**
Center_age^a^ SRH					0.01 (0.00–0.01)	**0.005**
BIC/AIC	6559.9/6474.9		6583.8/6480.5		6583.7/6468.4	
LR test^c^				0.947		**0.005**
Marginal/Conditional R^2^	0.240/0.774		0.239/0.774		0.243/0.775	

The bold values indicate the results are statistically significant (p < 0.05).

a Center_age, center age at visit on 60; MKE = Milwaukee; MSN, Madison; WRAT3, wide range achievement test (third edition); College, education years ≥ 16, *APOE*, apolipoprotein E; SRH, self rated health; z-comorbidities, z-scores of number of co-morbidities; z-num_prescriptions, z-scores of number of prescriptions; BIC, bayesian information criterion; AIC, akaike information criterion; LR, likelihood ratio. Male, Madison, Non-white and No college are reference levels. p-value (< 0.05) indicates that you can reject the null hypothesis that the coefficient is equal to 0.

b Model 1: covariates + random effects; Model 2: Model 1 + SRH, z-num_prescriptions, z-comorbiditites; Model 3: Model 2 + significant interactions (age and z-num_prescriptions, age and SRH).

c Test for the model 1 vs model 2, model 1 vs model 3. p-value (< 0.05) means adding the variables together explained a significant amount of variability in the outcome.

Table 8 summarizes the results of Models 1-3 for delayed recall. Younger participants, female, higher WRAT-3 reading scores, or having a college degree were significantly associated with a higher delayed recall. Although one significant interaction between age and co-morbidities were retained in model 3, all model fit statistics indicated that model 2 and 3 did not account for significantly more variability in delayed recall than model 1.

**TABLE 8:** The associations between medications, co-morbidities and delayed recall z-scores.

Predictors	Model 1^b^	Model 2^b^			Model 3^b^	
	β (95%CI)	p	β (95%CI)	p	β (95%CI)	p
Center_age^a^	−0.05 (−0.06–−0.04)	**<0.001**	−0.05 (−0.06–−0.04)	**<0.001**	−0.05 (−0.06–−0.04)	**<0.001**
Female	0.47 (0.35–0.59)	**<0.001**	0.47 (0.36–0.59)	**<0.001**	0.47 (0.36–0.59)	**<0.001**
White	0.21 (−0.16–0.57)	0.271	0.20 (−0.16–0.57)	0.277	0.21 (−0.16–0.57)	0.262
LaCrosse	0.01 (−0.12–0.14)	0.906	0.01 (−0.12–0.14)	0.926	0.01 (−0.12–0.14)	0.919
MKE	−0.28 (−0.63–0.08)	0.123	−0.27 (−0.62–0.09)	0.143	−0.27 (−0.62–0.08)	0.136
WRAT-III Reading	0.03 (0.02–0.04)	**<0.001**	0.03 (0.02–0.04)	**<0.001**	0.03 (0.02–0.04)	**<0.001**
College	0.24 (0.11–0.36)	**<0.001**	0.24 (0.11–0.36)	**<0.001**	0.24 (0.11–0.37)	**<0.001**
*APOE* ε4 carriers	−0.08 (−0.19–0.03)	0.147	−0.08 (−0.19–0.03)	0.156	−0.08 (−0.19–0.03)	0.151
Practice	0.17 (0.14–0.20)	**<0.001**	0.17 (0.14–0.20)	**<0.001**	0.17 (0.14–0.20)	**<0.001**
SRH			−0.003 (−0.04–0.04)	0.868	-0.003 (−0.04–0.04)	0.889
z-comorbidities			−0.0001 (−0.05–0.05)	0.995	0.02 (−0.03–0.07)	0.446
z-num_prescriptions			−0.02 (−0.06–0.02)	0.258	−0.02 (−0.06–0.02)	0.24
Center_age^a^ z-comorbiditites					−0.01 (−0.01–−0.00)	**0.023**
BIC/AIC	6532.4/6447.4		6555.2/6452.0		6558.1/6448.8	
LR test^c^				0.703		0.160
Marginal/Conditional R^2^	0.214/0.792		0.214/0.792		0.216/0.792	

The bold values indicate the results are statistically significant (p < 0.05).

a Center_age, center age at visit on 60; MKE, Milwaukee; MSN, Madison; WRAT3, wide range achievement test (third edition); College, education years ≥ 16, *APOE*, apolipoprotein E; SRH, self rated health; z-comorbidities, z-scores of number of co-morbidities; z-num_prescriptions, z-scores of number of prescriptions; BIC, bayesian information criterion; AIC, akaike information criterion; LR, likelihood ratio. Male, Madison, Non-white and No college are reference levels. p-value (< 0.05) indicates that you can reject the null hypothesis that the coefficient is equal to 0.

b Model 1: covariates + random effects; Model 2: Model 1 + SRH, z-num_prescriptions, z-comorbiditites; Model 3: Model 2 + significant interactions between age and z-comorbiditites.

c Test for the model 1 vs model 2, model 1 vs model 3. p-value (< 0.05) means adding the variables together explained a significant amount of variability in the outcome.

**FIGURE 4:**
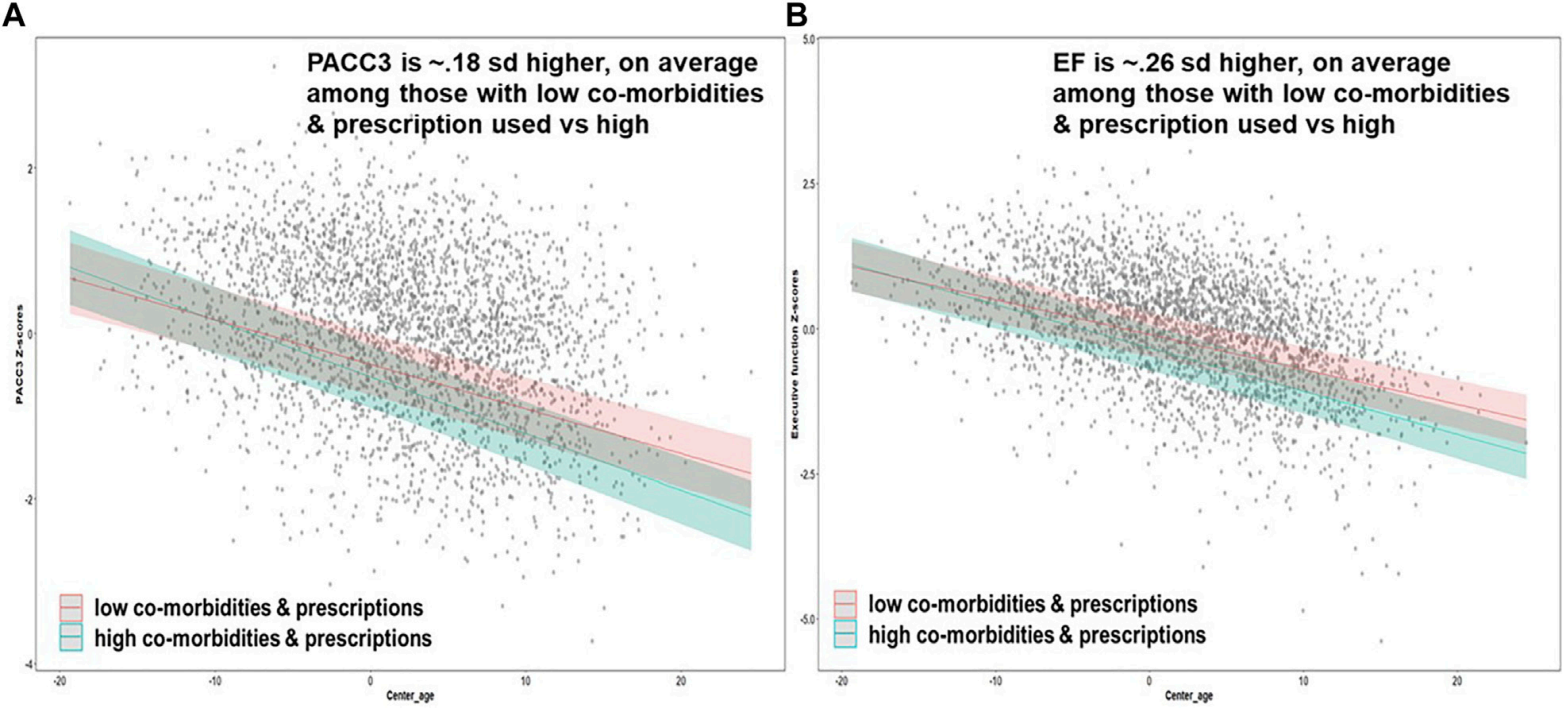
Predicted PACC scores and EF z-scores for those with different prescriptions and co-morbidities percentiles across the age range of the WRAP sample. The low (10^th^ centile) prescriptions (z-score = -1.02) corresponds to 0 prescriptions, the high (90^th^ centile) prescriptions (z-score = 1.31) corresponds to 6 prescriptions. The low (10^th^ centile) co-morbidities (z-score = -1.06) corresponds to 1 co-morbidities, the high (90^th^ centile) of co-morbidities (z-score = 1.33) corresponds to 7 co-morbidities. The gray dot is the observed scores. Holding other parameters constant: Male, White, Madison, WRATIII = 108, college degree = Yes, *APOE* ε4 carriers = Yes, SRH=3.7, Practice = 2.6 and the interaction term = −0.01 [age*co-morbidities for **(A)**, age*prescriptions for **(B)**].

Figure 4 depicts predicted EF z-scores for those with low prescriptions and co-morbidities across the age range of the WRAP sample using betas in Tables 4, 5 (estimating PACC3 and EF, respectively, across all ages holding other parameters constant. Using z-scores of 10th and 90th centile with model parameters, those with low co-morbidities and medications performed an average of 0.26 SD higher on EF than those elevated on both, and an average of 0.18 SD higher on PACC3 than those elevated on both.

### Sex Differences in the Associations Between Polypharmacy, Co-Morbidities, SRH, and Longitudinal Cognitive Performance

There were no significant three-way interactions across longitudinal cognitive composite domains. Three two-way interaction terms (sex*comorbidities, centered age*sex, centered age* comorbidities) were retained in the model for delayed recall. The model estimates were shown in Supplementary Figure S2A. Simple age-related delayed recall slopes indicated that the high comorbidity z-score group (90th percentile) declined faster than the low group (10th percentile), and females declined slower than males (Supplementary Figure S2B).

## Discussion

In this large prospective study (*n* = 1039) of cognitively unimpaired (at baseline) late middle age participants, we evaluated longitudinal outcomes in several cognitive domains that are sensitive to early cognitive decline. These include a global cognitive composite PACC3 (Mormino et al., 2017; Jonaitis et al., 2019), immediate learning, delayed recall, and executive function. At the baseline cognitive assessment, men and women differed across composite measures of immediate learning, delayed recall, executive function, and PACC3. Polypharmacy groups differed on executive function after adjusting age, sex, and WRAT-reading, but did not differ on race, education years, and APOE ε4 carriers. Also, pairwise comparisons showed age, sex, depression, co-morbidities, LiBRA indices, and SRH differences in the polypharmacy compared to the no polypharmacy group. This indicated that more medications are associated with worsened overall health. The most striking findings in this study were the association of the number of prescribed medications with PACC3, and the association of comorbidities with executive function in an AD risk-enriched cohort that was unimpaired at baseline. In the polypharmacy group, 6.2% progressed to clinical impairment at last visit. The patterns indicated that cognition in people with more co-morbidities or more prescribed medications declined faster, and cognitive declines in this sample may indicate increased dementia risk.

Several studies have reported the prevalence of polypharmacy in older people with dementia, e.g., associations between polypharmacy and cognitive impairments in patients with incident dementia (Soysal et al., 2019), impact of polypharmacy on progression of dementia (Zgheib et al., 2018), and prevalence of polypharmacy in people with dementia versus without dementia (Clague et al., 2017). Very few studies, however, have extended this line of research to participants who at baseline were cognitively unimpaired, late-middle-aged at-risk adults. Using a comprehensive approach, we examined the associations among polypharmacy, co-morbidities, and cognitive trajectories in a baseline unimpaired Alzheimer’s disease (AD) risk-enriched cohort. This study revealed that the prevalence of polypharmacy increased 17.4% during the follow-up period; this is similar to other studies that have found polypharmacy to be common in older people (Andersen et al., 2011; Rawle et al., 2018), higher than a study in a relatively healthy sample cohort (12.3%) (Khezrian et al., 2019), but much lower than the study of nursing home residents with advanced cognitive impairment (13.9 vs 2.89% on 10 or more regular prescribed medications) (Onder et al., 2013). Women took more medications than men in our study. Several studies have documented a higher prevalence of polypharmacy in women than men (Johnell et al., 2007; Venturini et al., 2011; Assari and Bazargan 2019a). Also, inappropriate prescription medications use is more common in women (Johnell et al., 2007; Johnell et al., 2009). Compared to men, women report more non-fatal chronic diseases (Murtagh and Hubert, 2004) and are more likely to seek care and take medications for such conditions. Women recognize (Vlahiotis et al., 2010) and communicate (Braybrook et al., 2011) their symptoms to their physicians and healthcare providers better than men. The same-sex differences are shown in low socioeconomic position older adults (Imran et al., 2009). However, a lower risk of polypharmacy for women than men was found in (Assari and Bazargan, 2019a), the reason may be due to better adherence of women to prescriptions. Race does not differ on polypharmacy, similar to the study in (Assari and Bazargan, 2019b). Our study found consistent evidence of associations between polypharmacy and cognitive decline, but the association is lower than other studies (Khezrian et al., 2019; Umegaki et al., 2019; Assari et al., 2020). Possible reasons include that there were only medication and morbidities assessed at a single time point (Khezrian et al., 2019), cross-sectional study design, and use of only an individual cognitive test score or a single cognitive domain (Umegaki et al., 2019). In contrast, in an Austrian study polypharmacy was not linked to cognitive decline; however, hyper-polypharmacy was positively associated with low cognitive performance (Alzner et al., 2016). One reason our study identified declines associated with polypharmacy when Alzner et al. did not may be that WRAP’s cognitive composites are more sensitive to preclinical decline than outcomes analyzed by Alzner et al. The association between polypharmacy and cognition in our study is similar to findings from a UK investigation (Rawle et al., 2018). Importantly, these associations remained significant after adjusting for comorbidities and other covariates, supporting the view that the risk effect of polypharmacy on cognitive decline and neurodegeneration does not solely occur via mechanisms related to comorbidities risk.

### Strengths, Limitations, and Future Directions

The strengths of this study include evaluation of longitudinal outcomes across several cognitive domains including those that are sensitive to early cognitive decline, specifically: a global cognitive composite [PACC3 (Donohue et al., 2014; Jonaitis et al., 2019)], immediate memory, delayed memory, and executive function (Clark et al., 2016), all of which provide more detailed outcomes for analyses than are used in routine health record databases. In our sample, detailed information allowed us to control for possible determinants of polypharmacy, such as education and disease burden (Imran et al., 2009), that might serve to confound associations with cognitive outcomes. The current findings indicate that the number of prescribed medications is associated with decreased PACC3 in an AD risk-enriched cohort that was unimpaired at baseline, after controlling for comorbidities and other possible confounding variables. Future research will study the specific types of medications that are linked to more severe cognitive decline.

Limitations include the following. First, our convenience sample and recruitment age range resulted in a sample that may not yield generalizable results. For example, the WRAP sample is limited in racial diversity and it might be too young to see clinically meaningful associations between prescribed medication numbers, co-morbidities, and some cognition composite scores. For instance, while a study conducted in Europe and Israel found a significantly greater decline in cognitive function (Vetrano et al., 2018), this group was considerably older (mean age at follow-up = 84) than our WRAP sample (mean age at follow-up = 65.6). Second, we did not examine whether or not prescribed medication numbers and co-morbidities had a negative effect on Alzheimer’s disease biomarkers including beta-amyloid plaques, phosphorylated tau, and neurodegeneration (Jack et al., 2018); this will be an important area of future study in the WRAP cohort. One study has shown the use of medications with medium or high anticholinergic activity was associated with neuroimaging biomarkers of brain hypometabolism and atrophy (Risacher et al., 2016). Additionally, our recent study found that some morbidities moderated the relationship between cognitive decline and amyloid burden (Clark et al., 2019). These results suggest that amyloid burden or other AD biomarkers may be an important variable to consider when assessing interactions between prescribed medication numbers, co-morbidities, and longitudinal cognitive trajectories in late middle-age. Also, we only measured the quantity, not the quality, of polypharmacy. Without knowing which cases of polypharmacy are inappropriate or appropriate, drug-drug interactions (DDIs), and drug-gene interactions (DGIs) (Keine et al., 2019), it is difficult to apply the results to health promotion and prevention of polypharmacy and cognitive function. Future research should explore whether things such as the type of medication, inappropriate polypharmacy, and medication interactions may reduce cognitive function and examine how the predictors in this paper are associated with incident dementia or other neurological disorders. Another limitation of our study is that we do not document whether participants are taking medications as prescribed or not. In this study, we did not find significant 3-way interactions and a larger dataset may be needed to study the association between polypharmacy, comorbidities and cognitions by sex. A further limitation of the study concerns missing data.

In conclusion, we found that the number of self-reported prescribed medications were significantly associated with lower longitudinal PACC3 score, and co-morbidities were significantly associated with lower EF in a late-middle-aged, cognitively unimpaired at baseline cohort at increased risk for AD after adjusting for potentially confounding factors. The results may help with health promotion, including reducing potentially inappropriate polypharmacy, and may inform the design of dementia and MCI prevention programs by collecting the medication and co-morbidities information. Future work should evaluate whether this translates to an increased risk of reaching clinical levels of impairment earlier than healthier, less medicated peers.

## Data Availability

Data from the WRAP study are available through a data request process described here: https://wrap.wisc.edu/data-requests/.
