# Supplementary material for: Prescription Medications and Co-Morbidities in Late Middle-Age are Associated with Greater Cognitive Declines: Results from WRAP
Source: Front Aging. 2022 Jan 3;2:759695. doi: 10.3389/fragi.2021.759695 (PMC9261362; doi:10.3389/fragi.2021.759695)
Supplement: Supplementary file 1 [file Table1.docx]

Table S1: Self-reported Co-morbidities, n(%) reported at any study visit

| N | 1039 |  |  |
| --- | --- | --- | --- |
| Hypertension (n(%)) | 275 (26.5) | Migraine headaches (n(%)) | 260 (25.0) |
| Diabetes (n(%)) | 70 ( 6.7) | Other neuro disorder (n(%)) | 67 ( 6.4) |
| High Cholesterol (n(%)) | 487 (46.9) | Neurosurgery (n(%)) | 11 ( 1.1) |
| Depression (n(%)) | 301 (29.0) | Arthritis (n(%)) | 203 (19.5) |
| Anxiety (n(%)) | 145 (14.0) | Thyroid (n(%)) | 63 ( 6.1) |
| Schizophrenia (n(%)) | 3 ( 0.3) | Cancer (n(%)) | 90 ( 8.7) |
| Bipolar (n(%)) | 6 ( 0.6) | Heart Diease (n(%)) | 208 (20.0) |
| Other mental (n(%)) | 45 ( 4.3) | Sleep disorder (n(%)) | 130 (12.5) |
| Headinjury (n(%)) | 187 (18.0) | Lung Diease (n(%)) | 182 (17.5) |
| Unconscious (n(%)) | 209 (20.1) | Liver Disease (n(%)) | 20 ( 1.9) |
| Epilepsy/Seizures (n(%)) | 26 ( 2.5) | Kidney Disease (n(%)) | 24 ( 2.3) |
| Meningitis (n(%)) | 20 ( 1.9) | AIDS (n(%)) | 3 ( 0.3) |
| Stroke (n(%)) | 10 ( 1.0) | Other Major (n(%)) | 108 (10.4) |
| Parkinsons (n(%)) | 3 ( 0.3) | Vision (n(%)) | 130 (12.5) |
| Transient ischemic attack (n(%)) | 12 ( 1.2) | Hearing Loss (n(%)) | 68 ( 6.5) |
| Multiple sclerosis (n(%)) | 7 ( 0.7) |  |  |
| Co-morbidities Sum* (mean (sd)) | 3.25 (2.36) | Prescription Sum (mean (sd)) | 2.43 (2.52) |

* Participants could report up to 31 co-morbidities and 15 prescription medications.
