# Supplementary material for: Prescription Medications and Co-Morbidities in Late Middle-Age are Associated with Greater Cognitive Declines: Results from WRAP
Source: Front Aging. 2022 Jan 3;2:759695. doi: 10.3389/fragi.2021.759695 (PMC9261362; doi:10.3389/fragi.2021.759695)
Supplement: Supplementary file 2 [file Image2.pdf]

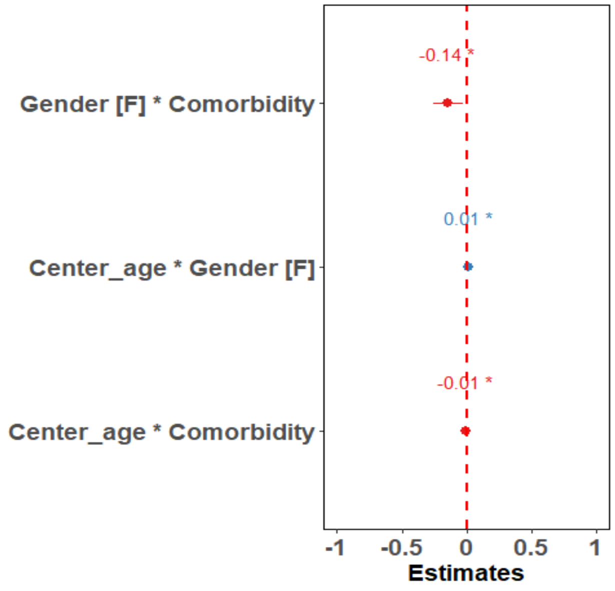

(a)

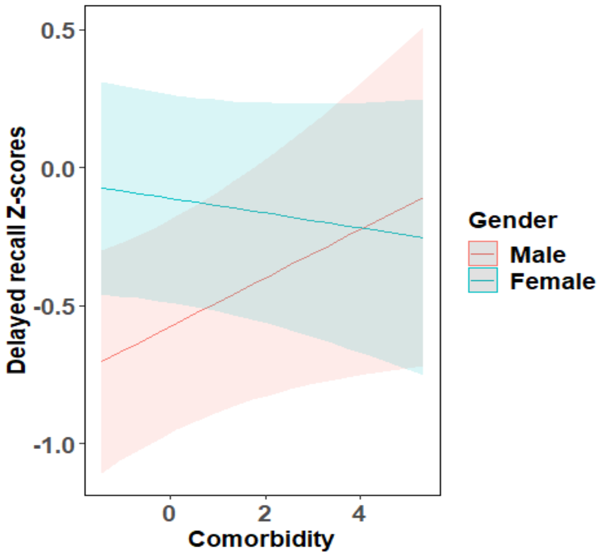

(b)

Figure S2a: Delayed recall: Model averaged regression interaction terms coefficient estimate and 95% confidence intervals. Figure S2b: The interaction plot between the number of comorbidities and sex on delayed recall. *p-value < 0.05.
