# Supplementary material for: Prescription Medications and Co-Morbidities in Late Middle-Age are Associated with Greater Cognitive Declines: Results from WRAP
Source: Front Aging. 2022 Jan 3;2:759695. doi: 10.3389/fragi.2021.759695 (PMC9261362; doi:10.3389/fragi.2021.759695)
Supplement: Supplementary file 3 [file Image1.pdf]

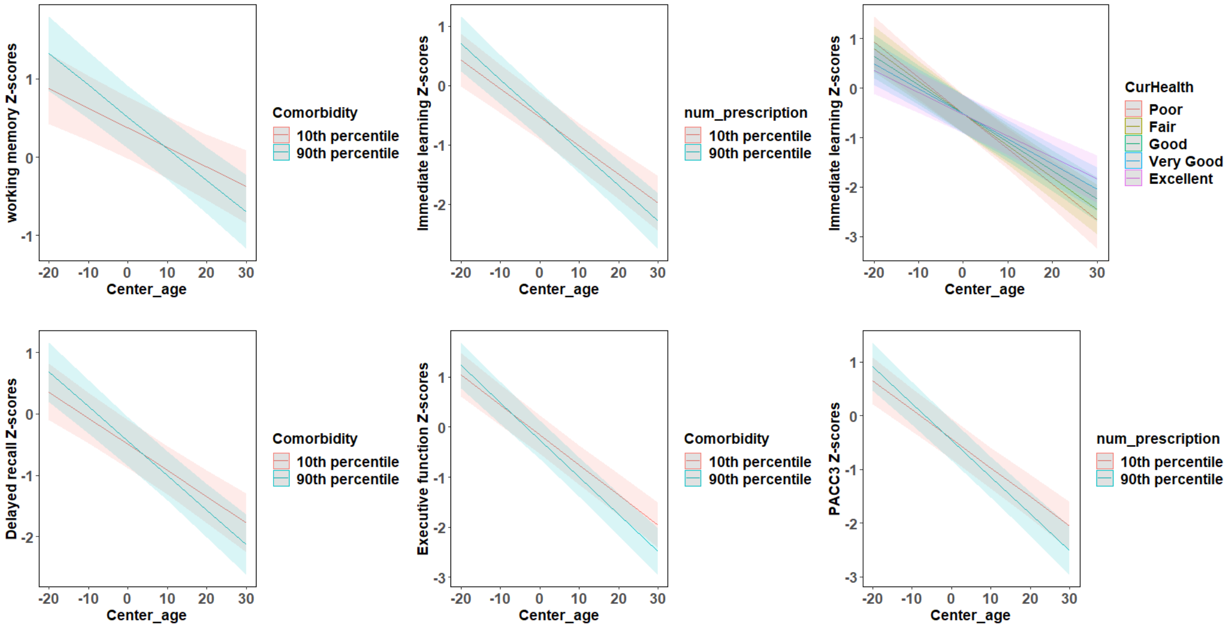

Figure S1: The interaction plot between key predictors and age on cognitive composite scores. Figure S1 legend: The 10th centile of prescriptions (z-score = -1.02) corresponds to 0 prescriptions, the 90th centile of prescriptions (z-score = 1.31) corresponds to 6 prescriptions. The 10th centile of co-morbidities (z-score = -1.06) corresponds to 1 co-morbidities, the 90th centile of co-morbidities (z-score = 1.33) corresponds to 7 co-morbidities.
